# Genotype-Phenotype Correlations of Pathogenic *COCH* Variants in DFNA9: A HuGE Systematic Review and Audiometric Meta-Analysis

**DOI:** 10.3390/biom12020220

**Published:** 2022-01-27

**Authors:** Sybren M. M. Robijn, Jeroen J. Smits, Kadriye Sezer, Patrick L. M. Huygen, Andy J. Beynon, Erwin van Wijk, Hannie Kremer, Erik de Vrieze, Cornelis P. Lanting, Ronald J. E. Pennings

**Affiliations:** 1 Department of Otorhinolaryngology, Hearing & Genes, Radboud University Medical Center, 6525 GA Nijmegen, The Netherlands; sybren.robijn@radboudumc.nl (S.M.M.R.); jeroen.smits@radboudumc.nl (J.J.S.); kadriye-sezer@live.nl (K.S.); patrick.huygen@radboudumc.nl (P.L.M.H.); andy.beynon@radboudumc.nl (A.J.B.); erwin.vanwyk@radboudumc.nl (E.v.W.); erik.devrieze@radboudumc.nl (E.d.V.); cris.lanting@radboudumc.nl (C.P.L.); 2 Donders Institute for Brain, Cognition and Behaviour, Radboud University Medical Center, 6500 GL Nijmegen, The Netherlands; hannie.kremer@radboudumc.nl; 3 Department of Human Genetics, Radboud University Medical Center, 6525 GA Nijmegen, The Netherlands

**Keywords:** DFNA9, *COCH*, genotype-phenotype correlations, systematic review, autosomal dominant hearing loss, vestibular diseases

## Abstract

Pathogenic missense variants in *COCH* are associated with DFNA9, an autosomal dominantly inherited type of progressive sensorineural hearing loss with or without vestibular dysfunction. This study is a comprehensive overview of genotype-phenotype correlations using the PRISMA and HuGENet guidelines. Study characteristics, risk of bias, genotyping and data on the self-reported age of onset, symptoms of vestibular dysfunction, normative test results for vestibular function, and results of audiovestibular examinations were extracted for each underlying pathogenic *COCH* variant. The literature search yielded 48 studies describing the audiovestibular phenotypes of 27 DFNA9-associated variants in *COCH*. Subsequently, meta-analysis of audiometric data was performed by constructing age-related typical audiograms and by performing non-linear regression analyses on the age of onset and progression of hearing loss. Significant differences were found between the calculated ages of onset and progression of the audiovestibular phenotypes of subjects with pathogenic variants affecting either the LCCL domain of cochlin or the vWFA2 and Ivd1 domains. We conclude that the audiovestibular phenotypes associated with DFNA9 are highly variable. Variants affecting the LCCL domain of cochlin generally lead to more progression of hearing loss when compared to variants affecting the other domains. This review serves as a reference for prospective natural history studies in anticipation of mutation-specific therapeutic interventions.

## 1. Introduction

Hearing loss (HL) is one of the most common sensory impairments and is estimated to affect 432 million people worldwide [1]. Congenital or childhood-onset sensorineural HL is caused by genetic conditions in more than half of the cases. However, adult-onset HL is much more complex and is caused by a combination of environmental and genetic (risk) factors [2]. Despite this, a growing number of dominantly inherited types of HL is associated with an adult-onset: e.g., DFNA10 (*EYA4* MIM: 601316), DFNA15 (*POU3F4*, MIM: 602459), DFNA21 (*RIPOR2*, MIM: 607017), and DFNA22 (*MYO6*, MIM: 606346) [3,4,5,6].

DFNA9 (MIM: 601396) is another example of an adult-onset type of dominantly inherited HL and is caused by missense variants in *COCH*. It is characterized by progressive high-frequency HL, often accompanied by a variable degree of vestibular dysfunction. In contrast, homozygous loss of function mutations in *COCH* lead to autosomal recessive, early-onset, and severe hearing loss (DFNB110) [7]. The exact prevalence of pathogenic missense variants in *COCH* is unknown, but DFNA9 has been reported in numerous families on four continents [8]. More than 25 different *COCH* variants have been reported to be causative of DFNA9 (Human Gene Mutation Database, April 2020). *COCH* encodes cochlin, which is expressed in, among other tissues, the human inner ear [9]. Cochlin is an extracellular matrix protein, but its exact role in the inner ear remains elusive. The protein is predicted to contain the following functional domains: an LCCL domain (Limulus factor C, Cochlin, and Late gestation lung protein Lgl1), two vWFA domains (von Willebrand Factor A-like 1 and 2), and two Ivd domains (short intervening domains 1 and 2). Several studies have shown that the LCCL domain of cochlin plays a role in the local innate immune system of the cochlea [10,11,12]. Based on these findings, it was hypothesized that the inner ear of subjects with DFNA9 might be more susceptible to infections [11].

Many studies have investigated the role of DFNA9-associated mutations in post-translational processing and cleavage of cochlin [13]. Several studies have suggested that variants affecting the individual cochlin domains lead to distinctly different phenotypes [8,13]. However, a concise, systematic review and meta-analysis of all pathogenic *COCH* variants and their corresponding phenotypes are still missing. This study aims to fill that gap and presents a systematic review of current DFNA9 literature and a meta-analysis of the available audiometric data to formulate robust genotype-phenotype correlations for DFNA9 to improve variant-specific counseling. The results from this study are also essential to define outcome measures that evaluate the effectiveness of future variant-specific (genetic) therapies.

## 2. Materials and Methods

### 2.1. Systematic Review

The Cochrane Handbook [14], the Centre for Reviews and Disseminations guidance for undertaking reviews in health care [15], HuGENet recommendations for systematic reviews [16], and the PRISMA statement [17] were applied in the review process. A review protocol was created and prospectively registered in PROSPERO (registration number CRD42018108199).

**Figure 1:**
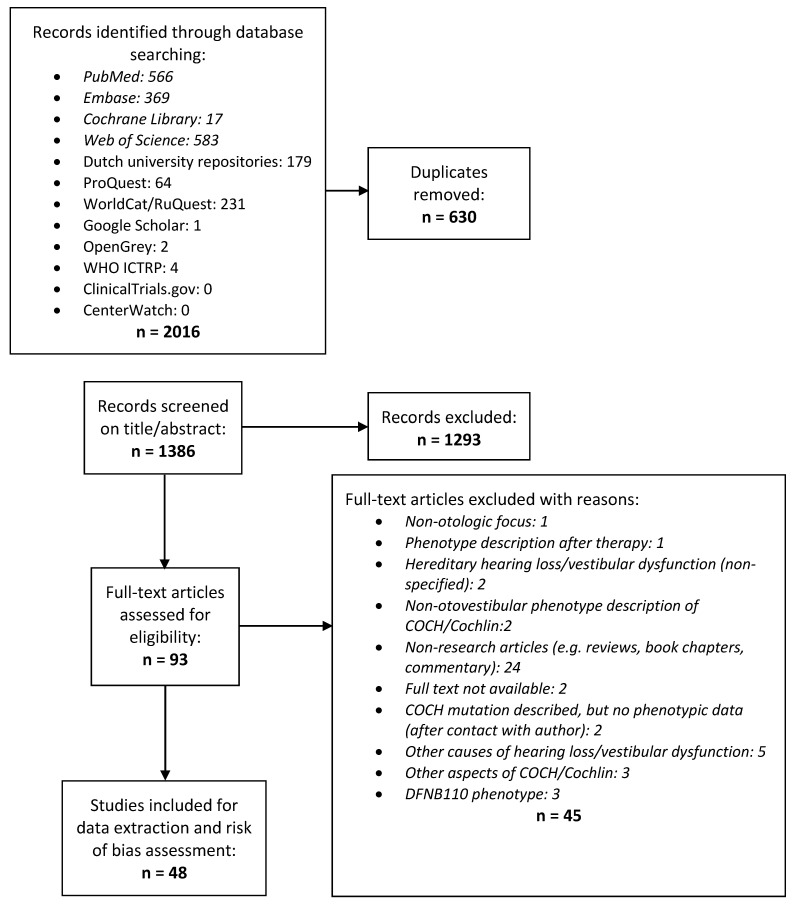
Flowchart of literature search. Detailing the systematic identification and inclusion of relevant studies on genotype-phenotype correlations in DFNA9, according to the preferred reporting items for systematic reviews and meta-analysis (PRISMA) statement.

A search was conducted in relevant databases (PubMed, NCBI’s Gene database, EMBASE, the Cochrane Library and Web of Science) focusing on DFNA9 genotype-phenotype correlation studies (last search update 20 January 2021). For a comprehensive search of ‘*COCH*’ and ‘DFNA9’, all available MeSH terms were combined with free text words of all known synonyms: ‘*COCH*’, ‘Cochlin’, ‘coagulation factor C homolog’, ‘Coch5B2’, ‘*COCH*-5B2’, and ‘DFNA9’ (Table S1). In addition, grey literature was searched to identify relevant missing reports. No restrictions were applied during the search process. After duplicate removal, the remaining studies were screened by title and abstract. Reference lists of these publications were scanned forward and backward for additional reports. Figure 1 shows a flowchart of the selection and filtering process. Two reviewers independently performed title and abstract screening, full-text screening, and final selection of eligible studies. Only studies published in either English or Dutch were included; however, none of the excluded studies in other languages appeared to contain audiovestibular examinations of DFNA9 patients. Studies on animal models were excluded. No restrictions were placed on the methods that evaluated the genotype, phenotype, sample size, or publication date. After each phase of screening, the reviewers met to reach a consensus.

Both reviewers used standardized forms (available upon request) to extract data and assess the risk of bias for each included study. Data on the self-reported age of onset, symptoms of vestibular dysfunction, and results of audiovestibular examinations were extracted for each underlying pathogenic *COCH* variant (Table S2). When available, normative test results for vestibular function (i.e., normal, hypofunction, or areflexia) were extracted. These were based on rotary chair tests, calorics, and video Head Impulse Tests (vHIT). Only data of subjects with a genetically confirmed DFNA9 diagnosis were included in this study. Corresponding authors were contacted to supply missing data. In addition, data were combined and deduplicated when multiple studies described the same individuals. Finally, disagreements were resolved by discussion and consultation of a third and fourth reviewer. 

Some of the previous studies reported phenotypic differences between variants affecting different domains of cochlin [8,13]. To further assess this, subjects were pooled based on their identified pathogenic variant into three different cochlin domain groups: the LCCL, Ivd1, and vWFA2 domain groups. RefSeq NM_004086.2 was used to describe all *COCH* variants.

### 2.2. Meta-Analysis

Original audiometric data were extracted and retrieved from the forty-eight included studies in the systematic review and used in the meta-analysis. Eleven of the included studies were performed on families previously diagnosed in our hospital [18,19,20,21,22,23,24,25,26,27]. We did not add additional hearing-impaired family members not previously reported in literature. Cross-sectional linear regression analyses were performed on bilateral averaged hearing thresholds to evaluate the progression of HL. For each variant, Age-Related Typical Audiograms (ARTA) were constructed to visualize progression of hearing loss [20,28]. ARTA were only constructed for a *COCH* variant when individual audiograms of at least eight different subjects of different ages were available. 

To assess potential differences in the progression of HL, the annual threshold deterioration (ATD) across the different *COCH* variants was calculated using cross-sectional non-linear regression analyses of hearing thresholds as a function of age. We first determined the pure-tone average across 0.5–4.0 kHz (PTA_0.5–4 kHz_) for each subject. A two-parameter logistic function was used to fit the data, i.e., ${\mathrm{PTA}}_{0.5-4\mathrm{kHz}}\;\sim \frac{130}{1+e^{\left(-\mathrm{scale}*\;\left(\mathrm{age}-{\mathrm{age}}_{\mathrm{mid}}\right)\right)}\;}$, similar to [29]. The parameter age_mid_ describes the value at which the hearing thresholds are halfway between 0 dB and the asymptotic value (fixed at 130 dB), and scale represents the slope of the function at this midpoint, which we use as an estimate for the ATD.

In contrast to the self-reported age of onset recorded in the systematic review, we also calculated the age of onset in the meta-analysis. The calculated age of onset was defined as when the calculated hearing threshold exceeds 25 dB, as the world health organization classifies this as abnormal hearing, and subjects could benefit from amplification [1]. The calculated age of onset was determined by interpolating the function fit using the parameters age_mid_ and scale. 

The logistic function was fitted to the audiometric data per domain group. The regression coefficients and their confidence intervals were assessed to test for differences between domains. For this, we constructed a null model by pooling all data across the three domains and successively adding grouping parameters using analysis of variance (ANOVA) testing and comparing the conditional likelihoods with the null model. The calculated age of onset and the ATD were obtained for each domain. We further obtained a fit describing progression over time for each variant with at least five data points and assessed the 95% confidence intervals of the calculated age of onset and progression of HL. 

All analyses were conducted using R statistical programming language version 3.6.2 using packages dplyr, tidyr, nls, nls.multstart for analyses and data handling and ggplot2 for visualization. Code available at https://zenodo.org/badge/latestdoi/359572523 (accessed on 16 July 2021).

## 3. Results

### 3.1. Systematic Review

**Figure 2:**
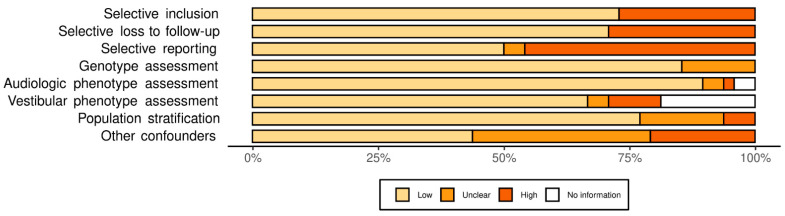
Risk of bias assessment. Review authors’ judgements about each risk of bias item presented as percentages across all included studies.

The total yield of the search strategy was 2016 studies. After full-text screening, 48 studies met the eligibility criteria and together reported on 27 different *COCH* variants (Figure 1 and Figure S1). Thirty-seven of these studies were retrospective family studies, describing the (audiovestibular) phenotype of *COCH* variants [18,19,20,21,22,23,24,25,26,27,29,30,31,32,33,34,35,36,37,38,39,40,41,42,43,44,45,46,47,48,49,50,51,52,53,54,55]. Eleven of the included studies were not primarily genotype-phenotype studies but validation studies of genetic analyses or histopathological studies [9,56,57,58,59,60,61,62,63,64,65]. Most of the selected studies were judged to have a high risk of bias on one or more domains (Figure 2 and Table S3), and only two studies had an overall low risk of bias [32,48]. Bias is mainly related to selective reporting and selective loss to follow-up. No restrictions were placed on the inclusion of articles based on bias. Articles describing homozygous loss-of-function variants in *COCH* that lead to DFNB110, [7,66,67,68] were excluded from further analysis; additional study details can be found in Table S4. We also decided to exclude the c.266C > A; p.(Pro89His) *COCH* variant, previously reported by Dodson et al., (2012), from this study (see Table S4). They identified this variant in a child who presented with congenital profound unilateral HL and an ipsilateral enlarged vestibular aqueduct (EVA) [69]. In contrast with the authors, we deem this variant non-pathogenic as an EVA has not been associated with DFNA9 in literature [34,43] and is a plausible explanation for the unilateral HL in this case. In addition, we identified this variant in two unrelated adult subjects with bilateral HL seen in our institute. In both cases, the variant segregated to a normal hearing parent (unpublished results). 

The 48 included studies reported on subjects predominantly from the Netherlands and Belgium, followed by the United States of America, China, Korea, Australia, Japan, Canada, Italy, Austria, Poland, and Hungary. Several studies reported on the same individuals or families, and deduplication was performed [9,18,19,20,21,23,29,33,35,37,42,56,59,61]. After deduplication and removal of subjects with HL but without a genetic confirmation of DFNA9, a total of 444 subjects with a genetically confirmed DFNA9 diagnosis were identified. An overview of extracted genotype and phenotype characteristics is given in Table S2.

**Figure 3:**
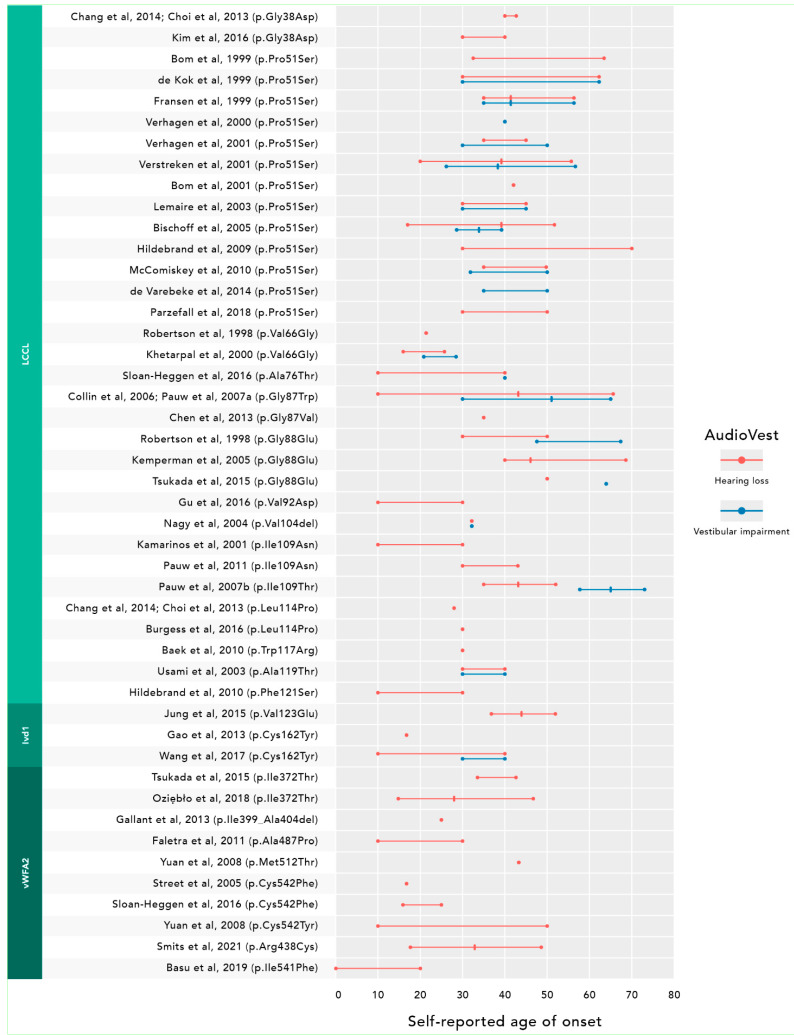
Self-reported age of onset of hearing loss and vestibular dysfunction. In total, 42 studies reported an age of onset of either hearing loss (red) or vestibular impairment (blue). Horizontal lines represent the reported range in decades, whereas a vertical line depicts means in years. The secondary *y*-axis shows the specific cochlin domains the variant affects.

Most studies (*n* = 46) used a self-reported age of onset of HL and/or vestibular symptoms (Figure 3). In general, HL presented with ages of onset that ranged from the 2nd to 7th decade. Four studies calculated the age of onset through (non)linear regression analyses on available audiometric results as an attempt to objectify this outcome (Table S2) [18,20,22,35]. Two of these studies, both on subjects with the p.(Pro51Ser variant), compared the self-reported ages of onset with those calculated by linear regression. Bom et al. (1999) described a self-reported age of onset ranging from 36 to 63 years and a calculated onset range of 34 to 51 years [18], whereas Bischoff et al. (2005) described a different family and reported these ranges as 18 to 51 and 38 to 49, respectively [22].

Twenty studies compared the age of onset of HL with the age of onset of vestibular impairment (Figure 3). Most of these studies (*n* = 12, among six on p.(Pro51Ser)) concluded that vestibular symptoms manifested at approximately the same age as HL. Eight studies presented a self-reported age of onset of vestibular dysfunction after the onset of HL, as was highlighted in a recent systematic review on subjects with the p.Pro51Ser variant (age of onset of HL: 32.8 years vs. vestibular dysfunction: 34–40 years) [70]. In contrast, Bischoff et al. (2005) measured vestibulo-ocular reflexes and concluded that vestibular dysfunction started on average nine years earlier than HL in subjects with this variant. Their study also showed vestibular function to deteriorate more rapidly over time than HL [22].

The presence or absence of vestibular symptoms was reported for 26 pathogenic variants. Vertigo was the most frequently mentioned symptom (for 50% of the variants), followed by instability and balance problems, especially in the dark (for 31%). A lack of vestibular symptoms was specifically reported (Table S2), two variants within the LCCL [30,58,59,60,61], one in the Ivd1 [64] and six in the vWFA2 domain [41,45,46,47,51,62]. 

All studies reported progression of HL over time. The ATD ranged from 0.7 dB to 7.0 dB/year, whereas the decrease in maximum speech recognition scores ranged from 0.99 to 3%/year (Table S2). The raw audiometric data were analyzed in more detail in the meta-analysis. The results of the vestibular examinations varied widely. We extracted 182 vestibular assessments from 160 individual subjects with 17 different pathogenic variants in *COCH*. Eighty subjects carried the p.(Pro51Ser) variant. It is important to note that the type and amount of vestibular function tests varied widely between studies (Table S2). Studies on subjects in the LCCL group reported more often vestibular dysfunction to occur in these subjects when compared to the subjects in the vWFA2 or Ivd1 groups (Table S2). Figure S2 shows the severity of vestibular dysfunction in the LCCL group as the percentage of subjects with either normal, hypofunction, or areflexia, as a function of the age in decade steps. No reliable comparison could be made with the other domain groups because of insufficient objective data in these groups. 

Some studies reported additional symptoms, including (incomplete segregation of) cardiovascular disease in two families with the p.(Pro51Ser) substitution [18,23], memory loss, and night blindness in a family with the p.(Phe121Ser) substitution [31]. In addition, Bischoff et al. (2007) investigated the presence of ocular abnormalities in DFNA9 subjects hailing from four different families [22,25,36,71] and concluded that the p.(Pro51Ser) and p.(Gly88Glu) variants were possibly associated with vertical corneal striae and related ocular symptoms as this phenomenon was present in 27 out of 61 affected subjects (and 5 out of 37 unaffected family members) [72]. Three other studies evaluated ocular involvement in 65 subjects with the p.(Pro51Ser) [37,50] and p.(Ile109Thr) [26] substitutions. In only one of these subjects, with the p.(Pro51Ser) substitution, a corneal scratch in one eye was found. Based on the latter studies, it can therefore be concluded that it is unlikely that vertical corneal striae are part of the DFNA9 phenotype. 

Twelve studies reported on temporal bone imaging of patients with DFNA9 [9,22,27,34,37,38,43,50,54,58,64,73]. Only two studies reported abnormal radiologic findings in subjects with the p.(Pro51Ser) substitution. Janssens de Varebeke et al. (2014) reported sclerotic lesions and/or narrowing of one or more semicircular canals on computed tomography (CT) and magnetic resonance imaging (MRI) (*n* = 8) [34], and Hildebrand et al., (2009) reported on the presence of bilateral superior semicircular canal dehiscence in one subject [43].

### 3.2. Meta-Analysis

**Figure 4:**
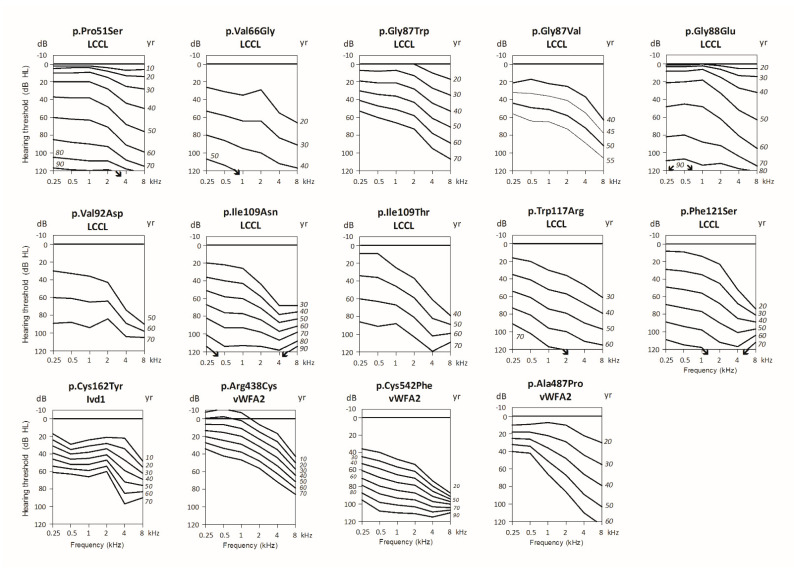
Age-Related Typical Audiograms (ARTA) for fourteen *COCH* mutations. ARTA are derived from cross-sectional linear regression analysis of last visit audiograms of affected subjects. Downward arrows indicate either out-of-scale measurements or underestimation of mean thresholds due to the exclusion of such measurements. Yr: age in years, dB HL: decibel hearing level, kHz: kilohertz.

Figure 4 shows the ARTA that was constructed for 14 *COCH* variants. This was impossible for the other variants due to an insufficient number of subjects for which audiometric data were available. In those cases, individual audiograms of the affected subjects are presented in Figure S3. Five variants (p.Val104del [44], p.Ala119Thr [38], p.Ile399_Ala404del [45], p.Met512Thr [41], p.Cys542Tyr [41]) were excluded from the meta-analysis and individual presentation (Figure S3) because of a lack of audiometric data, despite attempts to retrieve data via corresponding authors. In general, individual audiograms and ARTA showed progression of HL over all frequencies, although higher frequencies deteriorated earlier and more than lower frequencies (see Figure 4). 

A total of 744 audiograms, obtained in 297 subjects, could be extracted and were included in the analysis. The majority of the included subjects pertain to the LCCL group (*n* = 249). In contrast, only a relatively small number of subjects belong to the Ivd1 or vWFA2 group (*n* = 4 and 44, respectively). Single audiometric assessments were available in 163 subjects. For the remaining 134 subjects, two or more (repeated) measurements were extracted (Figure S4).

**Figure 5:**
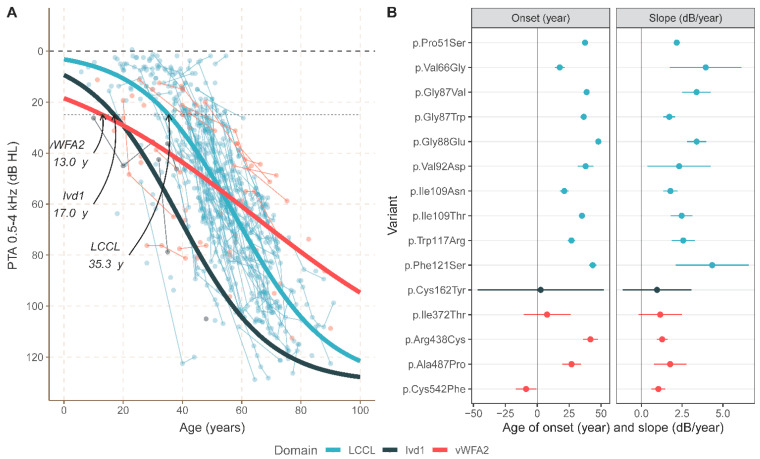
A phenotypic analysis of DFNA9; the calculated age of onset and annual threshold deterioration (ATD) across variants affecting cochlin within the LCCL, the Ivd1, and the vWFA2 domain. (**A**) ATD of the pure-tone average across PTA_0,5-4kHz_ with increasing age. Longitudinal data from single subjects are shown as a semi-transparent line between successive time points. A non-linear logistic fit shows the average PTA over time for the LCCL domain (blue), the Ivd1 domain (black), and the vWFA2 domain (red). (**B**) The parameter estimates and their 95% confidence intervals for the calculated age of onset (arbitrarily set at 25 dB) and the ATD (dB/year) for the various variants within the LCCL domain (blue), the vWFA2 domain (red), and the Ivd1 domain (black). The dashed line shows the averaged calculated onset and ATD across all variants (unweighted for differences between the number of subjects for each variant).

Figure 5A shows the progression of HL with increasing age for all included DFNA9 subjects across the three domain groups. An ANOVA on the models showed that the data are best modeled by adding both a grouping variable describing the midpoints for the three domains (F(2740) = 1.52 *p* = 0.02) and a separate variable for the ATD (F(2738) = 17.09, *p* < 0.001), each compared to the null-model. The parameter fits indicated a calculated age of onset of 35.3 years for the LCCL group, 17.0 years for the Ivd1 group, and 13.0 years for the vWFA2 group (see Figure 5A). ATD is visible in all groups, and the estimates vary between 2.06, 2.14, and 0.91 dB/year for the LCCL group, the Ivd1 group, and the vWFA2 group, respectively. There are apparent differences in the calculated ages of onset of HL and especially the ATD between subjects in the three affected domain groups (see also Figure S5). Subjects in the LCCL group had hearing thresholds varying from near-normal in the first two decades of life to moderate-to-severe around the fifth decade to severe-to-profound from the 7th decade onwards. Hearing thresholds in subjects in the vWFA2 and the Ivd1 group, in general, showed higher thresholds in the first two decades. Note, however, that not all variants within each domain share the same phenotype; for some variants in the vWFA2 domain, the (calculated) age of onset is higher, and the progression of HL over time is much more gradual than for the variants in the Ivd1 and the LCCL domain [27].

For 15 variants, we have obtained more than five data points in the analyses allowing for a variant-specific analysis of the age of onset and ATD. Figure 5B shows the parameter estimates for the calculated ages of onset (years) and ATD (dB/year) of these variants. Subjects in the vWFA2 domain group have a highly variable calculated age of onset. The variability for the calculated age of onset for variants in the LCCL group ranges between 17.6–47.9 years. There are limited data on the Ivd1 domain, but the calculated onset for the single variant with sufficient data is also much earlier than for those in the LCCL group.

Most data on the ATD are available for subjects in the LCCL domain group. The range varies considerably from 1.7–4.3 dB/year (see Figure 5B and the fits for the individual variants in Figure S5). The ATD in subjects in the Ivd1 group is similar to that of the LCCL group (Figure 5A, pooled across all variants). However, individual variants may exhibit a slower progression of HL (Figure 5B). Subjects in the vWFA2 group have, on average, a more gradual ATD, independent from the rather variable calculated age of onset in this group.

From this comprehensive analysis can be concluded that substantial phenotypic differences exist between the different *COCH* variants, both within and across domain groups (Figure 5B).

## 4. Discussion

The systematic review of this study provides a comprehensive overview of the audiovestibular phenotype of all (*n* = 27) currently (June 2021) known variants in *COCH* associated with DFNA9. Age-related typical audiograms were constructed for counseling purposes and display the progression of HL in decade steps per variant when sufficient data were available. A meta-analysis of crude audiometric data showed high phenotypic variability not only between individuals with different *COCH* variants but also between subjects with the same *COCH* variant. Furthermore, variants that affect the LCCL domain are associated with more progression of HL when compared to variants that affect the other cochlin domains. The results of the present study are also important because of recent promising developments in the evolving field of genetic therapies for hereditary hearing loss. The first preclinical study [74] on the development of such a therapy for DFNA9 was recently published. The results of the present meta-analysis can be used as a baseline to assess the efficacy of such novel therapeutics in future clinical trials.

The age of onset of HL and vestibular dysfunction in DFNA9 is variable and was assessed via self-reported history in the studies included in the systematic review and calculated from audiometric data in the meta-analysis. The self-reported ages of onset for HL ranged from the 2nd to 7th decade of life and the 3rd to 7th decade for vestibular complaints. A self-reported age of onset is prone to recall bias [75] and reporting the age of onset in ranges without an average, or in decades (for example, used by [54]), can lead to over-or underestimation of the actual age of onset. Calculating the age of onset via a simple two-parameter model is a more robust indicator of the actual average age of onset than taking the average self-reported age of onset. Given the rapid progression of hearing loss that has been described for several DFNA9 mutations, these calculated ages of onset may be essential to determine the optimal moment of starting a (genetic) therapeutic intervention that aims to delay or stop progression of hearing loss.

Different opinions can be found in literature on whether audiologic or vestibular signs manifest first in subjects with the most-studied pathogenic variant in *COCH*: p.(Pro51Ser). Janssens de Varebeke et al., (2019) showed that subjects with this variant initially presented audiovestibular symptoms, with high variability, between 30 and 50 years of age [70]. Based on vestibular evaluation, they calculated that vestibular deterioration starts at an average age of 36 years and that vestibular areflexia is always present at 60 years. Additionally, they found that HL began at 32.8 years, which is slightly earlier than the result from our analysis (37.4 years). Based on their results, they concluded that vestibular impairment starts later but progresses faster than HL [70]. Their study, however, contradicts the study by Bischoff et al., (2005), who analyzed objective audiovestibular test results of the largest group of subjects (*n* = 74) studied to date, all of which belong to a single Dutch DFNA9 family in which the p.(Pro51Ser) mutation was identified. Bischoff et al., (2005) showed that vestibular function deteriorated faster but also manifested earlier than HL [22,70]. Based on our systematic review and the analyses performed in the studies mentioned above, we conclude that there are insufficient reliable studies to draw firm conclusions on whether audiologic or vestibular signs manifest first.

Previous genotype-phenotype correlation studies showed that vestibular dysfunction is most often reported by subjects with variants that affect the LCCL domain. Studies that reported on variants that affect the other cochlin domains, in general, reported a lack of vestibular complaints in their subjects [41,45,46,47,51,62,63]. There are, however, also studies that explicitly reported an absence of complaints in the presence of vestibular areflexia [40,46]. The latter is most likely due to central vestibular compensation, which occurs when subjects use somatosensory and/or visual cues to reweigh their loss of vestibular function [24,41,47]. We have shown that HL in subjects in the vWFA2 domain group is slowly progressive, especially when compared to subjects in the LCCL domain group that experience much more progression of HL. A similarly slow deterioration of vestibular function in the subjects of the vWFA2 domain group might go unnoticed more frequently since vestibular compensation is easier for subjects with a slow deterioration of vestibular function when compared to those with a faster deterioration or sudden vestibular loss. We, therefore, conclude that vestibular history is an unreliable assessment of vestibular function and that it is essential to include objective measurements of vestibular function in genotype-phenotype correlation studies of any type of hereditary HL. In addition, it is also important to realize that a wide range of objective tests can be used to evaluate vestibular function and that, even when similar tests are used, local calibration or used instruments may vary between different centers. This makes it challenging to review test results from different studies systematically. We, therefore, decided to apply a normative approach to the test results in this study. Most of the included studies in this systematic review based their vestibular outcomes on calorization and/or rotary chair tests. These tests mainly assess lateral semicircular canal function and might therefore under- or overestimate the severity of vestibular dysfunction. To create extensive vestibular genotype-phenotype correlations in the future, we strongly advise testing both otolith organs by ocular and cervical Vestibular Evoked Myogenic Potential (VEMP) tests and testing the separate semicircular canals by video Head Impulse Tests (vHIT) to assess all parts of the vestibular organ.

The results of the present study support physicians in counseling of DFNA9 patients on audiovestibular progression over time. The developed variant-specific ARTA are a useful tool for this purpose. In many subjects with DFNA9, progression of HL may eventually necessitate cochlear implantation [76]. The quality of the ARTA depends on the number of subjects that can be included for each variant. This number varied highly in this study, from only one subject with the p.(Ala119Thr) variant to 208 subjects with the p.(Pro51Ser) variant. The latter variant is a well-studied founder mutation in the Dutch/Belgian population. The number of subjects with other pathogenic variants in *COCH*, and thus the amount of available phenotypic data, is much smaller. The audiometric evaluations of these subjects are therefore prone to selection bias, especially because inter-and intrafamilial variation is known to occur in DFNA9 [23,26,29,35,39,52]. In studies with large numbers of included subjects, as is the case for the p.(Pro51Ser) variant, the influence of selection bias decreases. Variation in the phenotype may also be explained by genetic modifiers or the cumulative contribution of environmental factors like the use of ototoxic medication, noise exposure, or chronic ear infections [1]. These confounders are usually not reported in the assessed studies but can significantly impact hearing and distort the clinical phenotype associated with *COCH* variants for which only a few subjects have been reported.

We observed, in line with the literature [13], the trend that progression of HL associated with variants within the LCCL domain group is faster than in the other domain groups. The age of onset, however, is highly variable between the cochlin domains, the individual *COCH* variants, and even within families. This study identified clear phenotypic outliers that question the previously suggested correlation between the phenotype and affected cochlin domain. Firstly, subjects with the p.(Pro51Ser) or the p.(Val66Gly) variant, both affecting the LCCL domain, have a markedly different calculated age of onset of HL. Secondly, patients with the p.(Arg438Cys) variant that affects the vWFA2 domain show a remarkably high age of onset of HL (41.6 years). In contrast, the HL associated with the p.(Val66Gly) variant that affects the LCCL domain has a relatively early calculated age of onset (17.6 years). Thirdly, although the HL associated with the p.(Cys542Phe) and the p.(Ala487Pro) variants is comparably progressive, the calculated age of onset is much lower for subjects having the p.(Cys542Phe) variant (Figure 5B).

Over the last two decades, several studies evaluated the effects of various DFNA9-associated *COCH* variants on intracellular protein transport, post-translational processing, secretion, and dimer/oligomer formation of mutant cochlin proteins [8,41,64,77,78,79]. In 2014, Bae et al. analyzed the molecular effects of 7 different DFNA9-associated mutations and observed a negative correlation between the amount of accumulated intracellular mutant cochlin in overexpression studies and the age of onset of HL. For this, they took an average onset based on the reported range (i.e., (max-min)/2) of the (often self-reported) age of onset in small groups of individuals with the same variant. They correlated an earlier age of onset of HL in individuals with DFNA9-associated variants within the vWFA2 domain to intracellular aggregate formation, affecting intracellular trafficking and secretion of cochlin [8]. Although we observe an on average similar trend, the phenotypic outliers that we describe in our study provide the first suggestion of a more complex genotype-phenotype correlation for DFNA9.

A recent review on DFNA9 pathophysiology by Verdoodt et al. [13] concluded that pathogenic variants affecting the LCCL domain do not appear to alter intracellular cochlin trafficking and secretion but instead lead to dimerization of cochlin proteins. While the term misfolding is often used for the formation of these dimers, there is no direct evidence that an alteration in folding of the mutant cochlin protein underlies the observed dimerization. We compared the calculated age of onset and progression of HL obtained in the meta-analysis with the previously published molecular consequences of cochlin mutations upon over-expression in cell lines (Table S5). The available in vitro data do not provide a satisfactory explanation of the observed phenotypic differences between variants within the same functional domain. For example, the earlier age of onset associated with the p.(Val66Gly) variant as compared to, e.g., p.(Pro51Ser) and p.(Gly88Glu) is not recapitulated by a more-severe molecular phenotype in in vitro studies. In addition, the most severe phenotypes were previously concluded to result from defective intracellular cochlin transport and secretion, leading to enhanced intracellular aggregate formation [13]. However, the p.(Cys542Phe) variant in the vWFA2 domain is associated with the earliest onset of all variants in the study, yet p.(Cys542Phe) cochlin appears to be properly secreted and does not display defective intracellular transport [47,64]. As such, our audiometric meta-analysis indicates a more-refined genotype-phenotype correlation for DFNA9 than previously determined based on overexpression of variant cochlin proteins. While the observed molecular defects upon overexpression of mutant cochlin can be an indicator of variant pathogenicity, in vitro overexpression studies lack sensitivity to predict how individual pathogenic variants in cochlin affect phenotypic outcomes of HL. The expression levels in such assays usually far exceed the physiological levels of cochlin expression. This is important since it is well known that overexpression of proteins can violate the balanced intracellular gene transcription and translation, thereby affecting protein folding, complex assembly, and downstream regulatory pathways. In other words, all of the molecular defects that were previously attributed to mutant cochlin, including defects in post-translational protein processing, protein misfolding, and associated defects in protein cleavage and secretion, have also been reported as so-called overexpression artifacts [80] and have potentially obscured the precise molecular genotype-phenotype correlation of DFNA9 mutations. Therefore, the observed discrepancies between the outcome of our meta-analysis, and the previously published molecular defects after overexpression of mutant cochlin proteins, advocate for more refined studies using physiological expression levels and advanced cell culture models or animal models. Only then will we be able to elucidate why some of the variants affecting the LCCL or the vWFA2 domains lead to a markedly different phenotype compared to the majority of variants affecting these domains.

## 5. Conclusions

This study presents an extensive overview of HL and vestibular dysfunction associated with different pathogenic variants in *COCH* and is vital for variant-specific counseling of individuals with DFNA9 and their relatives. Significant differences in both age of onset and progression of HL and vestibular dysfunction were seen between and within subjects with different pathogenic variants in *COCH*. In general, variants affecting the LCCL domain of cochlin lead to more progression of hearing loss when compared to variants affecting the other domains. The outcome of this study also emphasizes the need to further refine the molecular genotype-phenotype correlation of DFNA9. In addition, the results presented in this study also provide an essential step to define intervention criteria and endpoints for anticipated future clinical trials of novel (variant-specific) therapeutic interventions for DFNA9.

## Data Availability

Code and scripts used in the meta-analysis are published at https://zenodo.org/badge/latestdoi/359572523 (accessed on 16 July 2021). Additional data are available upon reasonable request.

## Supplementary Materials

The following supporting information can be downloaded at: https://www.mdpi.com/article/10.3390/biom12020220/s1, Table S1: Search strategy; Table S2: Study characteristics and overview of the audiovestibular phenotype; Table S3: Risk of bias summary; Table S4: Variants in *COCH* that are not associated with DFNA9; Table S5: Suspected mechanisms of action of different pathogenic *COCH* variants (based on literature) correlated to calculated age of onset and progression of HL; Figure S1: *COCH* domains (SP, LCCL, ivd1, vWFA1, ivd2 and vWFA2) and reported pathogenic variants causative for DFNA9; Figure S2: Vestibular function, based on objective vestibular examinations, over decades in percentage of subjects from the LCCL group; Figure S3: Individual audiograms at various ages of affected subjects of seven *COCH* variants; Figure S4: The distribution of the number of subjects per variant categorized by affected cochlin domain (LCCL, Ivd1 and vWFA2); Figure S5: Progression of hearing loss in subjects grouped by affected domain of cochlin and specific pathogenic variant.

## Abbreviations

HL	hearings loss
MIM	Mendelian Inheritance in Man number
DFNA	DeaFNess autosomal dominant
DFNB	DeaFNess autosomal recessive
vHIT	video Head Impulse Tests
ARTA	Age-Related Typical Audiograms
VEMP	Vestibular Evoked Myogenic Potential
ANOVA	analysis of variance
