# Supplementary material for: Genotype-Phenotype Correlations of Pathogenic COCH Variants in DFNA9: A HuGE Systematic Review and Audiometric Meta-Analysis
Source: Biomolecules. 2022 Jan 27;12(2):220. doi: 10.3390/biom12020220 (PMC8961530; doi:10.3390/biom12020220)
Supplement: Supplementary file 1 [file biomolecules-12-00220-s001.zip › biomolecules-1539304-supplementary/biomolecules-1539304-supplementary .pdf]

Genotype-phenotype correlations of pathogenic *COCH* variants in DFNA9: a HuGE systematic review and audiometric meta-analysis

Supplementary Information

Supplementary Table S1: Search strategy

**PubMed/Gene database**	"COCH protein, human" [Supplementary Concept] OR "Deafness, Autosomal Dominant 9" [Supplementary Concept]<br>OR<br>COCH [text word (tw)] OR cochlin [tw] OR coagulation factor C homolog [tw] OR Coch5B2 [tw] OR COCH-5B2 [tw] OR DFNA9 [tw]<br>OR<br>PubMed Links for Gene
**EMBASE**	Cochlin/<br>OR<br>(COCH OR cochlin OR coagulation factor C homolog OR Coch5B2 OR COCH-5B2 OR DFNA9).mp
**The Cochrane Library**	(Search "All text") COCH OR Cochlin OR "coagulation factor C homolog" OR Coch5B2 OR COCH-5B2 OR DFNA9
**Web of Science**	(Search on "Topic") COCH OR cochlin OR "Coagulation factor C homolog" OR Coch5B2 OR COCH-5B2 OR DFNA9

Legend: PubMed, NCBI's Gene database, EMBASE, the Cochrane Library and Web of Science were searched for relevant studies. All available MeSH terms were combined with free text words of all known synonyms of COCH and DFNA9.

Supplementary Table S2. Study characteristics and overview of the audiovestibular phenotype

Variant in COCH Protein domain	Author year	Population /ethnicity	Sample size		Clinical evaluation					Self-reported age of onset and complaints			
			Genetically confirmed (inheritance pattern)	Clinically suspected	Audiologic	Vestibular	Additional	Onset of hearing loss	Onset of vestibular complaint	Vestibular complaints	Vestibular results		
											Va	Vh	Vn
c.113G>A (p.Gly38Asp) LCCL	Wei et al, 2014	Chinese	13 (AD)	NA	History, PE, PTA	History, PE	NA	Late-onset	NA	No symptoms	NA	2	1
	Chang et al, 2014	Korean	1 (AD)	NA	History, PTA	History, VNG, caloric, rotatory	NA	41	NA	No symptoms			
	Choi et al, 2013	Korean	1 (AD)	NA	History, PTA	History, VNG, caloric, rotatory	NA	41	NA	No symptoms			
	Kim et al, 2016	Korean	6 (AD)	12	History, PTA	History, PE, caloric, rotatory, ECOG	NA	30-40	NA	No symptoms			
c. 151C>T (p.Pro51Ser) LCCL	Bom et al, 1999	Dutch	16 (AD)	6	History, PE, PTA	History, ENG, caloric, rotatory	History (CVD), PE (neurologic)	36–63 Calculated: 34–51	NA	NA	62	23	17
	de Kok et al, 1999	Dutch	23 (AD)	24	History, PE (otologic), PTA	History, EOG, rotatory	PE (neurologic)	30–62	30–62	Instability in the dark, oscillopsia			
	Bom etk al, 2003	Dutch	34 (AD)	NA	History, PTA	NA	NA	Calculated: 32,1–40,7	NA	NA			
	Fransen et al, 1999	Belgian	34 (AD)	13	History, PE, PTA	History, PE, vestibular testing (N/S)	NA	42 (35–56)	42 (35–56)	Vertigo/no symptoms			

Verstreken et al, 2001	Belgian	60 (AD)	13	History, PE, PTA, supraliminary tests*	History, ENG, caloric, rotatory	CT, MR, PE (neurologic, ophthalmic), urine sediment	39 ± 10 (20–56)	38 ± 11 (5–57)	Instability in the dark, vertigo, tendency to fall, drunken feeling, aural fullness
Janssens de Varebeke et al, 2014	Caucasian	9 (NA)	NA	History, PTA	History, ENG, caloric, rotatory	CT, MR, CBCT	46[a]	35–50	Instability in the dark, vertigo, oscillopsia, aural fullness, dizziness
Bom et al, 2001	Dutch Flemish	42 (AD)	NA	PTA, SA	NA	NA	42	NA	NA
Verhagen et al, 2000	NA	4 (AD)	NA	History, PTA	ENG, caloric rotatory	NA	Calculated: 28	40	Instability in the dark, vertigo, nausea, oscillopsia
Bischoff et al, 2005	Dutch	30 (AD)	14	History, PE, PTA, SA, BAEP	History, ENG, rotatory	CT, MR, PE (neurologic)	39 (18–51) Calculated: 43 (38–49)	34 (29–39)	Vertigo/no symptoms
Lemaire et al, 2003	Belgian	16 (AD)	NA	History. PE, PTA, SA	History, ENG, caloric rotatory	NA	30–45	30–45	Instability in the dark, vertigo, oscillopsia, aural fullness/no symptoms
Verhagen et al, 2001	Dutch	8 (AD)	8	History, PTA	History, ENG, caloric, rotatory	History (CVD)	35–45	4th–5th decade	Instability in the dark, vertigo, nausea, oscillopsia, motion sickness/no symptoms
Parzefall et al, 2018	Austrian	5 (AD)	2	History, PE (otologic), PTA, Freiburger-SA	NA	CT, MR	4th–5th decade	NA	NA
Hildebrand et al, 2009	American	6 (AD)	10	History, PTA	History	CT	4th–7th decade	NA	Instability in the dark, dizziness, no symptoms
McComiskey et al, 2010	Canadian	7 (AD)	5	History, PTA	History	History (ophthalmic), CT	35–49	32–50	Instability, vertigo, dizziness/no symptoms

	Alberts et al, 2018	Dutch	16 (AD)	NA	NA	Rotatory, VEMP, vHIT, velocity step tests, caloric	NA	NA	< 49[a]	NA			
c.197T>G (p.Val66Gly) LCCL	Robertson et al, 1998	American	16 (NA)	5	History, PE (ORL, genetic), PTA, SA	Vestibular tests(N/S)	PE (neurologic), MR	20.8	NA	No symptoms	3	NA	NA
	Khetarpal et al, 2000	American	3 (AD)	NA	History, PE (ORL)	History, ENG, SVAR, S/D posturography	PE (neurologic)	16–26	21–28	NA			
c.226G>A (p.Ala76Thr) LCCL	Sloan-Heggen et al, 2016	Caucasian	3 (AD)	NA	History, PE, PTA	History	NA	Teens - 40	40	Vertigo, dizziness, balance problems/no symptoms	NA	NA	NA
c.259G>T (p.Gly87Trp) LCCL	Collin et al, 2006	Dutch	1 (AD)	24	History, PTA	History, ENG	NA	43 (10–66)	51 (30–65)	Instability in the dark, vertigo, tendency to fall/no symptoms	2	8	NA
	Pauw et al, 2007a	Dutch	17 (AD)	8	History, PE, PTA, SA	History, ENG, caloric, rotatory	PE (ophthalmic)						
c.260G>T (p.Gly87Val) LCCL	Chen et al, 2013	Chinese	5 (AD)	3	History, PE, PTA, OAE	History, PE, ENG	NA	Mid–40s	NA	Vestibular symptoms	NA	1	NA
c.263G>A (p.Gly88Glu) LCCL	Kemperman et al, 2005	Dutch	16 (AD)	13	History, PE (otologic) PTA, SA	History, ENG, caloric, rotatory	History (CVD), CT, MR	46 (40–68)	Approximately the same age as HI	Instability in the dark, vertigo, tendency to fall/no symptoms	5	2	6
	Robertson et al, 1998	American	6 (AD)	6	History, PE (ORL, genetic), PTA, SA	Vestibular tests(N/S)	PE (neurologic), MR	4th decade–5th decade	48–67	Vertigo, oscillopsia, balance problems, dizziness/no symptoms			
	Tsukada et al, 2015	Japanese	4 (AD)	1	History, PTA, SA	History, caloric, VEMP, CDP	NA	Early 50s	64	Vestibular symptoms, dizziness/no symptoms			

c.275T>A (p.Val92Asp) LCCL	Gu et al, 2016	Chinese	7 (AD)	4	History, PE, PTA	History, ENG, caloric	NA	2nd–3rd decade	NA	Vertigo, dizziness/no symptoms	NA	5	NA
c.311_313delTAG (p.Val104del) LCCL	Nagy et al, 2005	Hungarian	1 (de novo or AD	NA	History, PTA	NA	NA	32	32	Vertigo, nausea, vomiting	1	NA	NA
c.326T>A (p.Ile109Asn) LCCL	Pauw et al, 2011	Australian	8 (AD)	6	History, PTA, SA	History	NA	30–43	After symptoms of HL	Instability in the dark, vertigo, oscillopsia, tendency to fall/no symptoms	NA	NA	NA
	Kamarinos et al, 2001	Australian	13 (AD)	NA	NA	NA	NA	2nd–3rd decade	By the time of profound HL	Instability in the dark, vertigo, oscillopsia, balance problems			
c.326T>C (p.Ile109Thr) LCCL	Pauw et al, 2007b	Dutch	11 (AD)	2	History, PE, PTA, SA	History, ENG, caloric, rotatory	PE (ophthalmic)	43 (35–52)	65 (58–73)	Instability in the dark, vertigo, oscillopsia, tendency to fall/no symptoms	NA	NA	6
	Burgess et al, 2016	American	1 (AD)	4	History, PTA	History	CT	30	NA	No symptoms			
c.341T>C (p.Leu114Pro) LCCL	Chang et al, 2014	Korean	1 (AD)	NA	History, PTA	History	NA	28	NA	No symptoms	NA	NA	NA
	Choi et al, 2013	Korean	1 (AD)	NA	History, PTA	History	NA	28	NA	No symptoms			
c.349T>C (p.Trp117Arg) LCCL	Baek et al, 2010***	Korean	10 (AD)	2	History, PE, PTA, SA	History, PE, caloric	NA	Early 30s	NA	Dizziness/no symptoms	1	NA	NA
c.355G>A (p.Ala119Thr) LCCL	Usami et al, 2003	Japanese	1 (AD)	1	History, PTA	History, caloric	CT	4th decade	4th decade	Vertigo, dizziness	NA	1	NA
c.362T>C (p.Phe121Ser) LCCL	Hildebrand et al, 2010	American	7 (AD)	3	PE (otologic & genetic), PTA	ENG, caloric	NA	2nd–3rd decade	NA	Vertigo, positional nystagmus, balance problems, dizziness	NA	NA	NA
c.368T>A (p.Val123Glu) Ivd1	Jung et al, 2015	Korean	3 (AD)	3	History, PTA, ABR	History, caloric	CT, MR	44.4 ± 6.3 (37–52)	NA	No symptoms	NA	NA	1
c.485G>A (p.Cys162Tyr) Ivd1	Kim et al, 2015	Korean	1 (AD)	5	History, PE (otologic	NA	NA	30[a]	NA	NA	NA	NA	NA

					& genetic), PTA								
	Wang et al, 2017	Chinese	2 (AD)	28	History, PTA, tympanometry, TEOAE, DPOAE	History, VNG, ECOG, caloric, VEMP, vHIT	NA	2nd–4th decade	4th decade	Vertigo, aural fullness/no symptoms			
	Gao et al, 2013	Chinese	9 (AD)	16	History, PTA	History	NA	17	NA	No symptoms			
	Kim et al, 2016	Korean	5 (AD)	1	PTA	History, PE, caloric, rotatory	NA	NA	NA	Vertigo, vomiting, aural fullness, dizziness			
c.1115T>C (p.Ile372Thr) vWFA2	Oziębło et al, 2018	Polish	5 (AD)	2	History, PTA	History, VEMP (cervical and ocular)	NA	28.25 years (15–47 years)	NA	No symptoms	2	1	1
	Tsukada et al, 2015	Japanese	3 (AD)	6	History, PTA, SA	History	NA	33-42	NA	No symptoms			
c.1312C>T (p.Arg438Cys) vWFA2	Smits et al, 2021	Dutch	14 (AD)	1	PTA, SA, History, otoscopy	History	MRI	33 (18–49)	NA	Balance problems	4	NA	10
c.1196_1213del18 (p.Ile399_Ala404del) vWFA2	Gallant et al, 2013	American	3 (AD)	5	History, PE	NA	MR	Mid to late 20s	NA	No symptoms	NA	NA	NA
c.1459C>G (p.Ala487Pro) vWFA2	Faletra et al, 2011	Italian	9 (AD)	1	History, PE (ORL), PTA	History, caloric	NA	2nd–3rd decade	NA	Vertigo, dizziness	NA	1	NA
c.1535T>C (p.Met512Thr) vWFA2	Yuan et al, 2008	Chinese	2 (AD)	NA	History, audiometric testing (N/S)	Oculomotor test, CDP, rotatory, SOT, VEMP	NA	43	NA	No symptoms	NA	NA	2
c.1580T>G (p.Phe527Cys) vWFA2	Cho et al, 2012	Korean	2 (AD)	4	History, PE, PTA	History, **PE (vestibular) posturography, rotatory	NA	26[a]	NA	No symptoms	NA	NA	1

c.1621A>T, (p.Ile541Phe) vWFA2	Basu et al, 2019	Caucasian	1 (AD)	3	History, otoscopy, PTA	NA	CT, autoimmune workup	Childhood	NA	NA	NA	NA	NA
c.1624T>C (p.Cys542Arg) vWFA2	Tsukada et al, 2015	Japanese	1 (AD)	3	History, PTA, SA	History	NA	Grade school	Grade school	Vertigo	NA	NA	NA
c. 1625G>A (p.Cys542Tyr) vWFA2	Yuan et al, 2008	Chinese	6 (AD)	NA	History, audiometric testing (N/S)	Oculomotor test, CDP, rotatory, SOT, VEMP	NA	2nd–5th decade	NA	No symptoms	NA	6	NA
c.1625G>T (p.Cys542Phe) vWFA2	Street et al, 2005	American	16 (AD)	4	History, PTA, tympanometry	History, oculomotor test (VNG), CDP, EOG, ENG, caloric, rotatory, VEMP	NA	16–25	NA	No symptoms	NA	3	NA
	Sloan-Heggen et al, 2016	Caucasian	3 (AD)	NA	History, PE, PTA	History	NA	NA	NA	NA			

Legend: COCH transcript NM_004086.2 was used as reference sequence. Vestibular function assessment (both examination and reports of subjects) varied between and within subjects. Therefore, all findings in a study are reported. NA: not available, AD: autosomal dominant [a]: derived from the audiogram, pure tone average or vestibular test(s), Vb: bilateral vestibular dysfunction, Vu: unilateral vestibular dysfunction, Vn: no vestibular dysfunction, PTA: pure tone audiometry, PE: physical examination, OAE: otoacoustic emission, SA: speech audiometry, BAEP: brainstem auditory evoked potential, supraliminary tests*: speech audiometry, tone-decay test, short increment sensitivity index test, impedance audiometry, transient evoked otoacoustic emissions & brainstem auditory responses, TEOAE: transient evoked product otoacoustic emission, DPOAE: distortion product otoacoustic emission, ABR: auditory brainstem response, ECOG: electrocochleography, ENG: electronystagmography, SVAR: sinusoidal vertical axis rotation, S/D posturography: static & dynamic posturography, CDP: computerized dynamic posturography, SOT: sinusoidal oscillation test, VEMP: vestibular evoked myogenic potential, EOG: electrooculography, VNG: videonystagmography, vHIT: video head impulse test, ** PE (vestibular: spontaneous nystagmus, head shaking test, Dix-Hallpike test, positional test), CT: computed tomography, MR: magnetic resonance, CBCT: cone beam computed tomography. ***One family (1St) described by Robertson et al. (1998), with the p.Trp117Arg variant, was excluded because of a lack of phenotypic data [9].

Supplementary Table S3: Risk of bias summary.

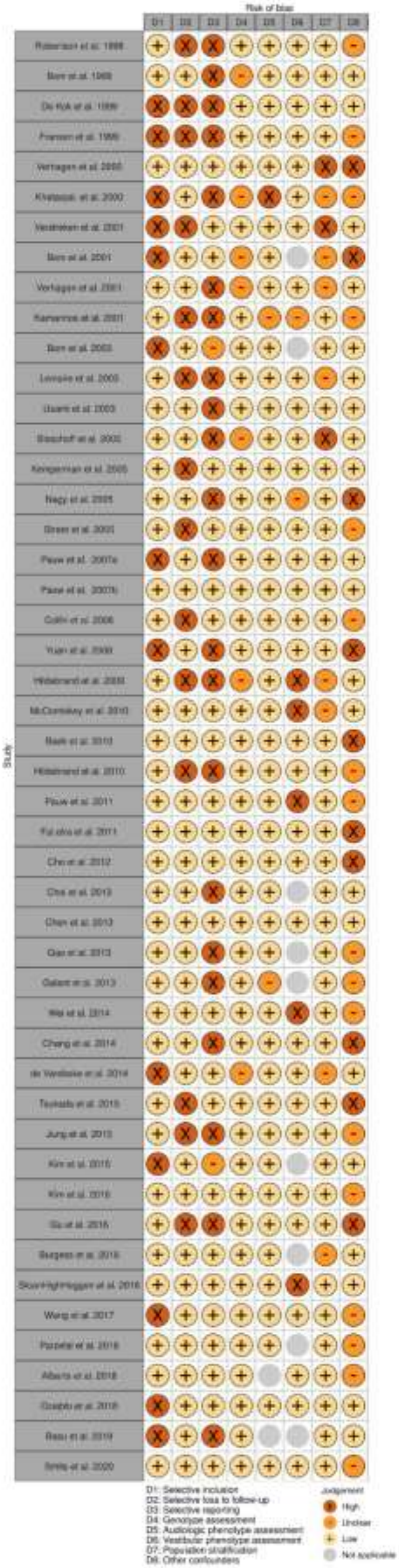

Legend: Judgements of two reviewers about each risk of bias items for all included studies according to the PRISMA guidelines

Supplementary Table S4: Variants in COCH that are not associated with DFNA9.

Variant (zygosity)	References	Sample size		Age of onset (in years)		Progression of hearing loss	Vestibular dysfunction		Remarks
		Genetically	Clinically	HL	V		Examination	Reported by subjects	
c.266C>A (p.Pro89His) (heterozygous)	Dodson et al. (2012)	1	NA	Birth	NA	NA	NA	NA	Enlarged vestibular Aquaduct; identified in two in-house normal hearing subjects
c.292C>T (p.Arg98X) (homozygous)	Janssens de Varebeke et al. (2018)	2	NA	Birth	First decade	1.8–2.0 dB/year	Vb / Vn	Vestibular symptoms/no symptoms	DFNB110 phenotype
c.116T>A (p.Leu39X) (homozygous)	Mehregan et al. (2019)	3	3	School age	NA	NA	NA	No symptoms	DFNB110 phenotype
c.984_985dup (p.Phe329Leufs*16) (homozygous)	Danial-Farran et al. (2020)	3	NA	Pre-lingual	NA	Yes	NA	NA	DFNB110 phenotype
c.631G>T (p.Glu211Ter) (homozygous)	Booth et al, (2020)	1	NA	Birth	NA	NA	NA	NA	DFNB110 phenotype
c.439A>T (p.Lys147Ter)* c.571_572delinsAG (p.Val191Arg) (compound heterozygous)	Booth et al, (2020)	1	NA	Birth	NA	NA	NA	NA	DFNB110 phenotype
c.271C>G (p.Arg91Gly) (homozygous)	Booth et al, (2020)	1	NA	Birth	NA	NA	NA	NA	DFNB110 phenotype
c.1093_1101del (p.Ser365_Asn367del) (homozygous)	Booth et al, (2020)	1	NA	Birth	NA	NA	NA	NA	DFNB110 phenotype

Legend: Study characteristics and overview of the audiovestibular phenotype of COCH variants not associated with DFNA9. COCH transcript NM_004086.2 was used as reference sequence. NR: not reported, HL: hearing loss, V: vestibular dysfunction, Vb: bilateral vestibular dysfunction, Vn: no vestibular dysfunction

Supplementary Table S5: Suspected mechanisms of action of different pathogenic COCH variants (based on literature) correlated to calculated age of onset and progression of HL.

Variant in Cochlin	Protein Domain	ER to Golgi transport	Cleavage	Secretion	Dimerisation /aggregation	Number of subjects in analysis (number of audiograms in analysis)	Calculated age of onset (years)	Progression (dB/year)
p.Pro51Ser	LCCL	normal	↓	normal	dimerization	130(397)	37,44	2,17
p.Val66Gly	LCCL	normal	↓	normal	dimerization	7(9)	17,63	3,95
p.Gly87Trp	LCCL	normal	N/A	normal	none	28(67)	36,43	1,71
p.Gly87Val	LCCL	normal	N/A	N/A	N/A	5(11)	38,75	3,39
p.Gly88Glu	LCCL	normal	↓ ↓	normal	dimerization	26(61)	47,88	3,39
p.Val92Asp	LCCL	normal	N/A	N/A	N/A	6(6)	37,92	2,32
p.Ile109Thr	LCCL	normal	↓ ↓ ↓	↓ ↓ ↓	dimerization	11(34)	35,06	2,48
p.Ile109Asn	LCCL	normal	N/A	N/A	N/A	8(52)	21,24	1,78
p.Trp117Arg	LCCL	normal	↓ ↓ ↓	normal	none	10(10)	26,79	2,57
p.Phe121Ser	LCCL	normal	N/A	N/A	N/A	10(18)	43,5	4,35
p.Cys162Tyr	Ivd1	ER-retention	↓ ↓ ↓	↓ ↓	oligomerization	3(7)	2,7	0,96
p.Ile372Thr	vWFA2	N/A	N/A	N/A	N/A	4(6)	7,7	1,16
p. Arg438Cys	vWFA2	N/A	N/A	N/A	N/A	14(29)	41,64	1,28
p.Ala487Pro	vWFA2	ER-retention	N/A	↓ ↓	oligomerization	9(9)	26,85	1,77
p.Cys542Phe	vWFA2	normal	N/A	normal	N/A	14(17)	-8,73	1,04

Legend: Table showing the effects of various DFNA9-associated COCH variants on intracellular protein transport, post-translational processing, secretion and dimer/oligomer formation of mutant cochlin proteins in relation to the calculated age of onset and annual progression. Different colors show the relative severity of the annual progression (last column) the calculated age of onset (second to last column), where green indicates slow progression of late onset, and red fast progression or early onset. NP_004077.1 was used as reference. N/A: not available. Arrows indicate amount of reduction in cochlin cleavage and secretion: ↓ = mild, ↓ ↓ = moderate, ↓ ↓ ↓ = severe.

Supplementary Figures

Supplementary Figure S1: COCH domains (SP, LCCL, ivd1, vWFA1, ivd2 and vWFA2) and reported pathogenic variants causative for DFNA9.

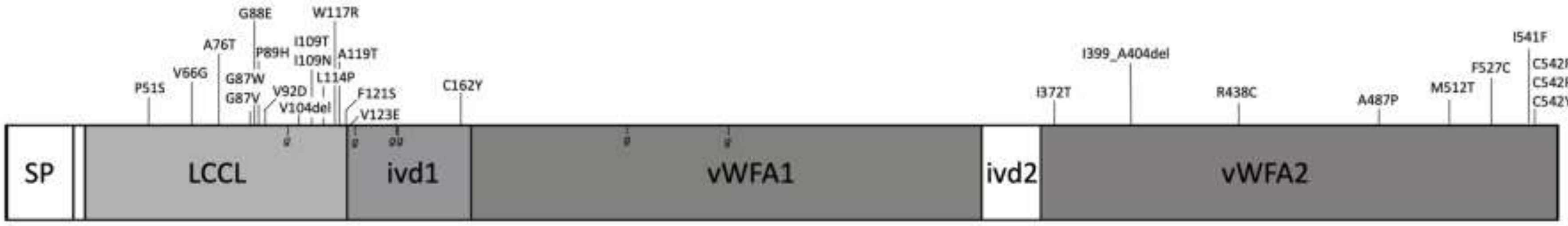

Legend: known and predicted glycosylation sites curated in the NextProt database are depicted as "g". Note that none of these sites are mutated in DFNA9." https://www.nextprot.org/entry/NX_O43405/sequence (accessed: 18 January 2022)

Supplementary Figure S2: Vestibular function, based on objective vestibular examinations, over decades in percentage of subjects from the LCCL group

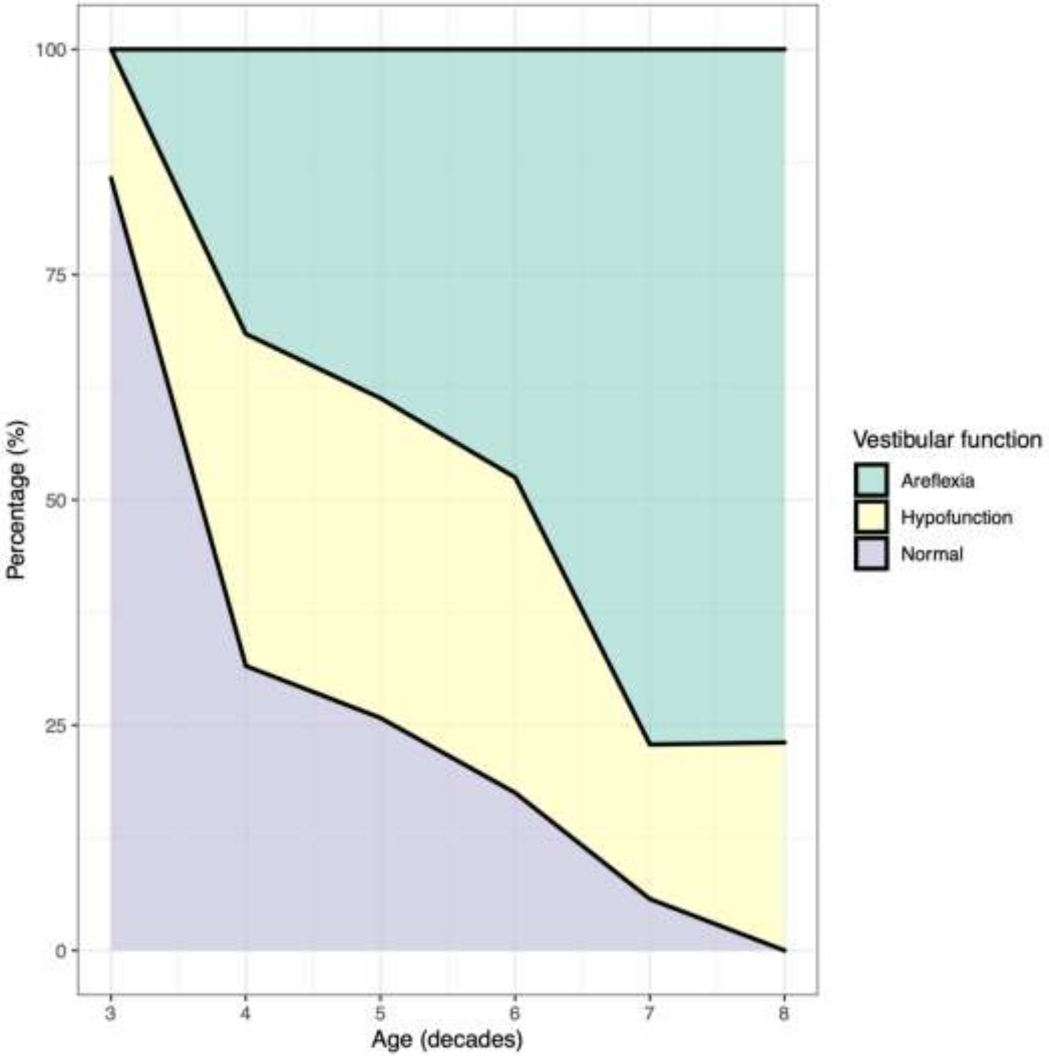

Legend: Figure showing the distribution of vestibular function at a specific age (Areflexia, Hypofunction, Normal) in percentages over time of patients with pathogenic variants affecting the LCCL domain.

Supplementary Figure S3: Individual audiograms at various ages of affected subjects of seven COCH variants

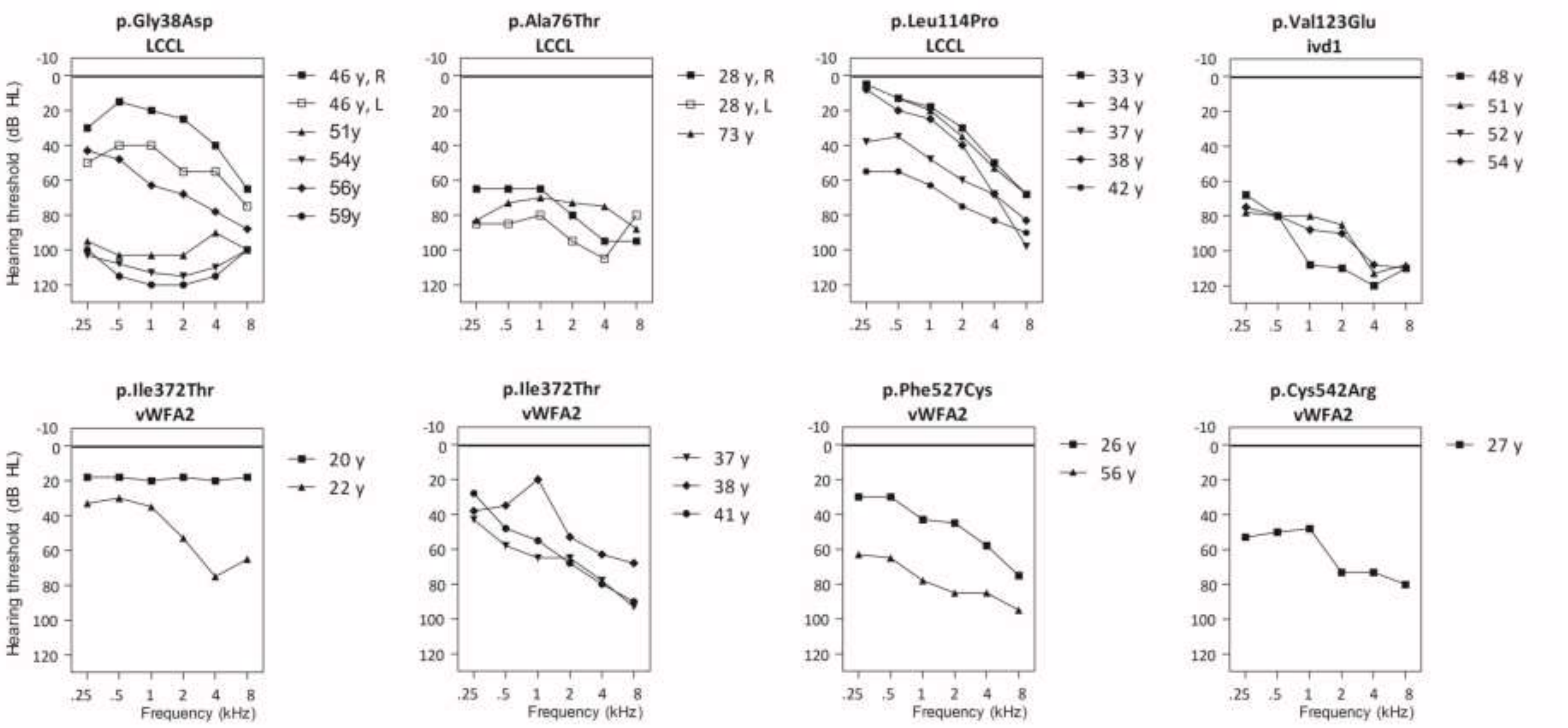

Legend: Air conduction threshold levels are depicted. In the case of asymmetry open symbols were used for the left ear. The audiometric data for p.Ile372Thr has been split in two panels for clarity purposes. R: right ear, L: left ear, dB HL: decibel hearing level, kHz: kilohertz, y: age in years

Supplementary Figure S4: The distribution of the number of subjects per variant categorized by affected cochlin domain (LCCL, Ivd1 and vWFA2).

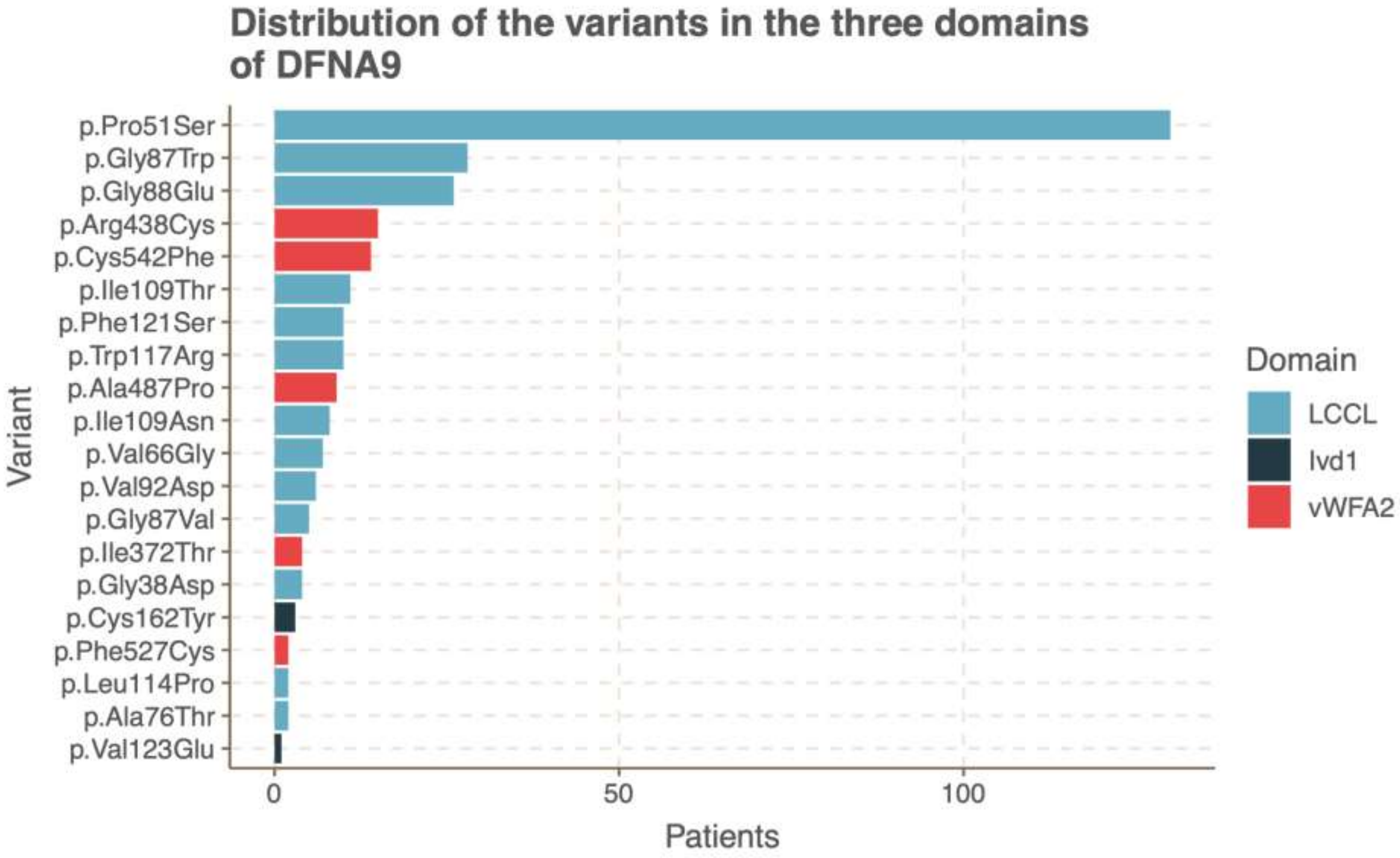

Legend: Figure showing the number of patients per variant and domain used in the meta-analysis.

Supplementary Figure S5: Progression of hearing loss in subjects grouped by affected domain of cochlin and specific pathogenic variant.

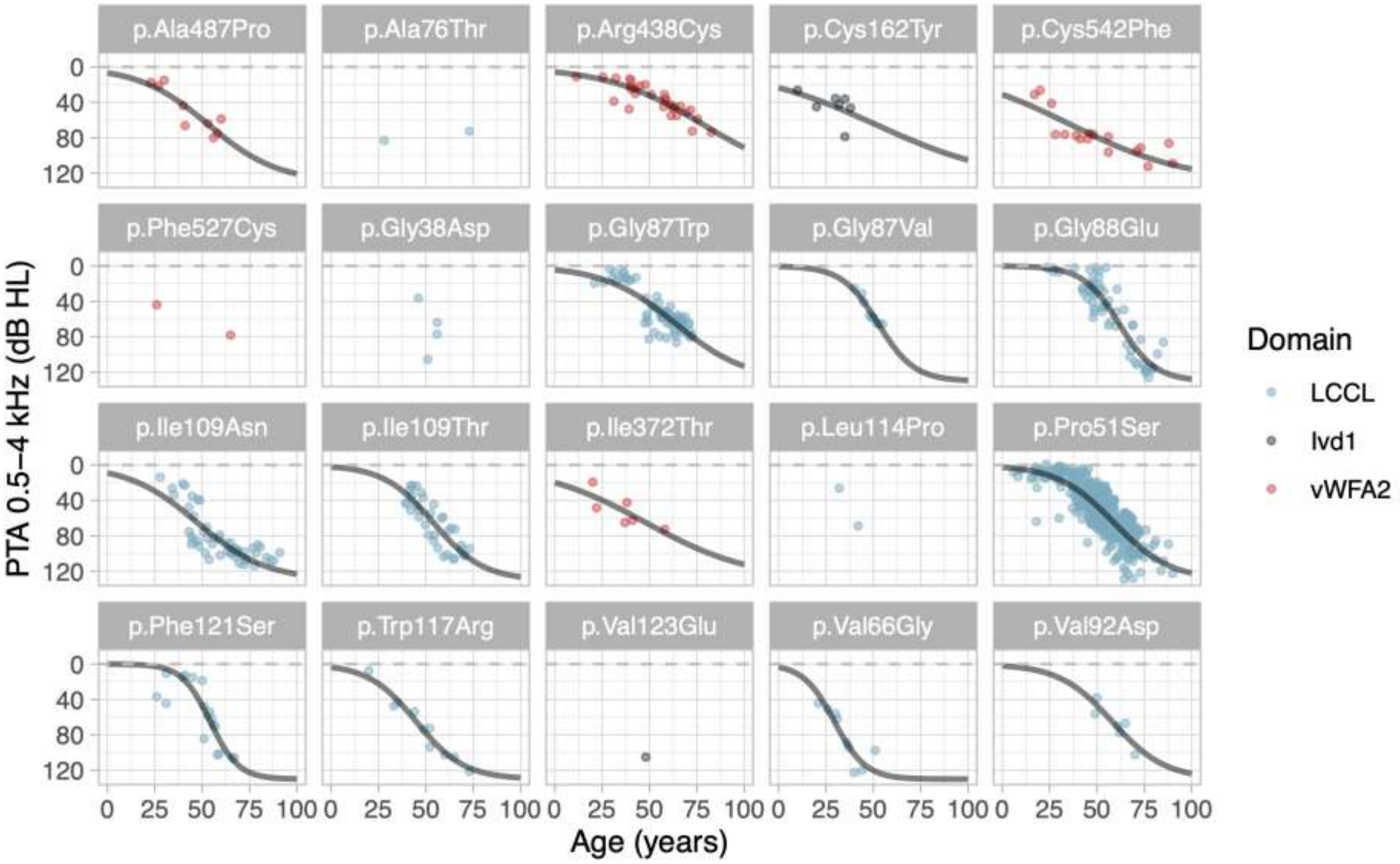

Legend: Figure showing the individual ATD of the pure-tone average across $PTA_{0,5\text{-}4kHz}$ with increasing age.
