# Supplementary material for: Genotype-Phenotype Correlations of Pathogenic COCH Variants in DFNA9: A HuGE Systematic Review and Audiometric Meta-Analysis
Source: Biomolecules. 2022 Jan 27;12(2):220. doi: 10.3390/biom12020220 (PMC8961530; doi:10.3390/biom12020220)
Supplement: Supplementary file 1 [file biomolecules-12-00220-s001.zip › biomolecules-1539304-supplementary/biomolecules-1539304-supplementary table S3.pdf]

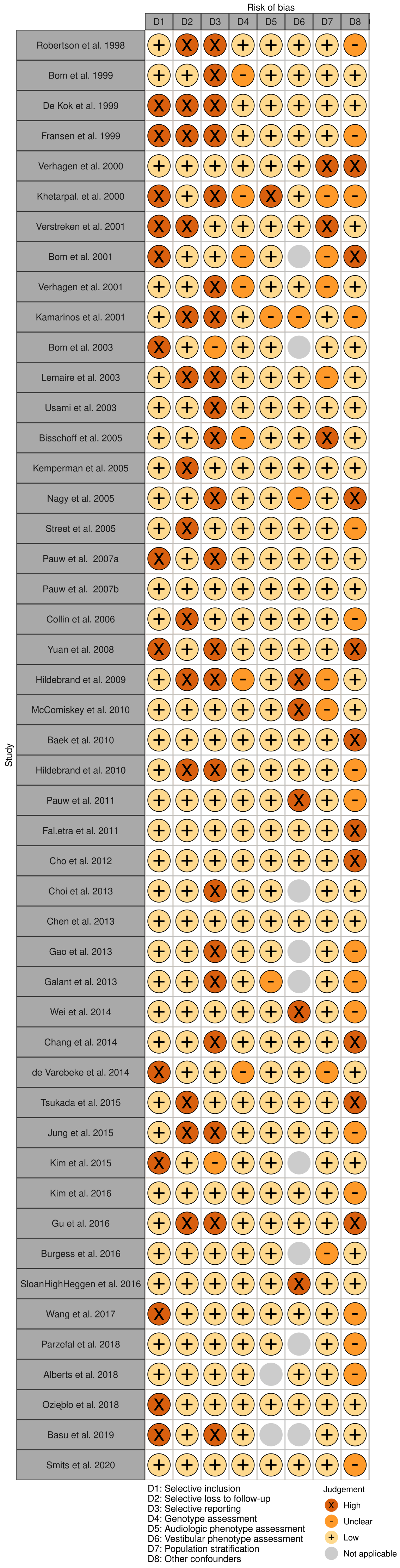
Risk of bias
D1
D2
D3
D4
D5
D6
D7
D8
Study
Robertson et al. 1998
Bom et al. 1999
De Kok et al. 1999
Fransen et al. 1999
Verhagen et al. 2000
Khetarpal. et al. 2000
Verstreken et al. 2001
Bom et al. 2001
Verhagen et al. 2001
Kamarinos et al. 2001
Bom et al. 2003
Lemaire et al. 2003
Usami et al. 2003
Bisschoff et al. 2005
Kemperman et al. 2005
Nagy et al. 2005
Street et al. 2005
Pauw et al. 2007a
Pauw et al. 2007b
Collin et al. 2006
Yuan et al. 2008
Hildebrand et al. 2009
McComiskey et al. 2010
Baek et al. 2010
Hildebrand et al. 2010
Pauw et al. 2011
Fal.etra et al. 2011
Cho et al. 2012
Choi et al. 2013
Chen et al. 2013
Gao et al. 2013
Galant et al. 2013
Wei et al. 2014
Chang et al. 2014
de Varebeke et al. 2014
Tsukada et al. 2015
Jung et al. 2015
Kim et al. 2015
Kim et al. 2016
Gu et al. 2016
Burgess et al. 2016
SloanHighHeggen et al. 2016
Wang et al. 2017
Parzefal et al. 2018
Alberts et al. 2018
Oziębło et al. 2018
Basu et al. 2019
Smits et al. 2020
D1: Selective inclusion
D2: Selective loss to follow-up
D3: Selective reporting
D4: Genotype assessment
D5: Audiologic phenotype assessment
D6: Vestibular phenotype assessment
D7: Population stratification
D8: Other confounders
Judgement
High
Unclear
Low
Not applicable
